# Evaluating the Accuracy of Morphological Identification of Larval Fishes by Applying DNA Barcoding

**DOI:** 10.1371/journal.pone.0053451

**Published:** 2013-01-31

**Authors:** Hui-Ling Ko, Yu-Tze Wang, Tai-Sheng Chiu, Ming-An Lee, Ming-Yih Leu, Kuang-Zong Chang, Wen-Yu Chen, Kwang-Tsao Shao

**Affiliations:** 1 Biodiversity Research Center, Academia Sinica, Taipei, Taiwan; 2 Fisheries Research Institute, Council of Agriculture, Keelung, Taiwan; 3 Institute of Zoology, College of Life Science, National Taiwan University, Taipei, Taiwan; 4 Center of Excellence for Marine Bioenvironment and Biotechnology, National Taiwan Ocean University, Keelung, Taiwan; 5 Department of Biology, National Museum of Marine Biology and Aquarium, Pingtung, Taiwan; University of Hamburg, Germany

## Abstract

Due to insufficient morphological diagnostic characters in larval fishes, it is easy to misidentify them and difficult to key to the genus or species level. The identification results from different laboratories are often inconsistent. This experiment aims to find out, by applying DNA barcoding, how inconsistent the identifications can be among larval fish taxonomists. One hundred morphotypes of larval fishes were chosen as test specimens. The fishes were collected with either larval fish nets or light traps in the northern, southern and northwestern waters of Taiwan. After their body lengths (SL) were measured and specimen photos were taken, all specimens were delivered, in turn, to five laboratories (A–E) in Taiwan to be identified independently. When all the results were collected, these specimens were then identified using COI barcoding. Out of a total of 100 specimens, 87 were identified to the family level, 79 to the genus level and 69 to the species level, based on the COI database currently available. The average accuracy rates of the five laboratories were quite low: 80.1% for the family level, 41.1% for the genus level, and 13.5% for the species level. If the results marked as “unidentified” were excluded from calculations, the rates went up to 75.4% and 43.7% for the genus and species levels, respectively. Thus, we suggest that larval fish identification should be more conservative; i.e., when in doubt, it is better to key only to the family and not to the genus or species level. As to the most misidentified families in our experiment, they were Sparidae, Scorpaenidae, Scombridae, Serranidae and Malacanthidae. On the other hand, *Mene maculata* and *Microcanthus strigatus* were all correctly identified to the species level because their larvae have distinct morphology. Nevertheless, barcoding remains one of the best methods to confirm species identification.

## Introduction

Correct identification of fish eggs and larval fishes to the species level can let us understand which species are spawning where and when, their hatching and nursery grounds, and their possible migration routes in their early life history. This information is very important for ecological monitoring, environmental impact assessment, fishery compensation, resource management, smuggling prevention and establishing marine protected areas [1], [2].

Traditionally, larval fish identification has always used morphological characters, such as the body shape, pigmentation, meristic count and measurements. However, these characters are not enough to identify every species, especially those rare and cryptic species [3]. In the early life history of fishes, many species share the same morphology, and their morphometric measurements are often duplicated [4]. In addition, the morphology of the same species can change quickly and significantly during its development from preflexion larvae to postflexion to the pre-juvenile stage, when the fish settles down. Thus, the same species at different developmental stages may be identified as a different species when using morphological characters. Consequently, using morphological characters to identify the species composition of larval fishes may not reflect the correct community changes. Furthermore, different larval fish taxonomists may have different capabilities and skills in identification, so even the same specimen can be identified inconsistently, which makes data comparison difficult.

Compare DNA barcoding with traditional morphology, morphology is poor in the discrimination among cryptic species of adult fish or larval fish while DNA barcoding maybe capable of species identification [5]. DNA barcode technique could be used as a rapid tool to survey many uncertain species, species composition, and cryptic species of adults in the study area [6]–[8] and distinguish morphologically similar species [9]. Shao et al. [10] has pointed out that only molecular identification can guarantee identification of fish eggs to the species level. We think this conclusion can be applied to larval fish identification, too. The application of DNA barcoding to larval fish identification has become popular in recent years [11]–[16]. Nevertheless, no paper has tried to use barcoding to evaluate the accuracy of morphologically identifying larval fishes.

This study attempts to do this evaluation by having different larval fish taxonomists identify the same larval fish specimens independently, using traditional morphological characters first. Then, we used DNA barcoding to identify their species names and calculate how accurate the different taxonomists were, and discuss some larval fish identification problems.

## Materials and Methods

### Fish Specimens

One hundred morphotypes of larval fishes were chosen as the experimental material. The definition of “larval fish” in this paper covers the developmental stages from preflexion to postflexion, including those “juvenile” in the process of settling down that were caught by the light trap. Afterward, their body length (SL) was measured and specimen photos were taken. All specimens were delivered in turn to five laboratories (A–E) in Taiwan for their independent morphological identification. The five laboratories were (not listed in order of A to E): (1) Biodiversity Research Center, Academia Sinica; (2) Institute of Zoology, National Taiwan University; (3) Fisheries Research Institute, Council of Agriculture; (4) Center of excellence for Marine Bioenvironment and Biotechnology, National Taiwan Ocean University; and (5) Department of Biology, National Museum of Marine Biology and Aquarium.

We used three different kinds of sampling tools to collect larval fishes for our surveys, including plankton net, light traps and Isaacs-Kidd midwater trawl (IKMT). All specimens were collected in the northern, southern and northwestern waters of Taiwan during the period between September 2006 and March 2008. All caught samples were preserved in 95% ethanol. To avoid damaging or destroying larval fish specimens when cutting the tissue for barcoding, 30 morphotypes with smaller body sizes have an additional specimen chosen as the “reference specimen,” which shares the most similar morphology and closest sampling time as its counterpart. The reason we use the term “reference” is because we cannot guarantee that the additional specimen is of the same species as the original based on morphology, unless verified by DNA barcoding. Because larval fish are still classified as zooplankton and no permit is needed for their collection. A total of 94 specimens with its sequences (74 species) was uploaded to the BOLD system (in Larval fishes from Taiwan project) the detail information was shown on Table S1.

### DNA Barcoding

DNA extracts were prepared from muscle tissue using the Genomic DNA Mini Kit. Approximately 650 bp were amplified from the 5′ region of the COI gene from mitochondrial DNA using the primers FishF1 and FishR1 [17]. The 25 µl PCR reaction mixes included 17.9 µl of ultrapure water, 2.5 µl of 10X PCR buffer, 0.3 µl of dNTP (40 mM), 1 µl of each primer (1 mM), 0.3 µl of Taq polymerase, and 2 µl of DNA template. The thermal regime consisted of an initial step of 4 min at 94°C followed by 32 cycles of 0.5 min at 94°C, 0.5 min at 50°C, and 1 min at 72°C, followed in turn by 9 min at 72°C, and then held at 12°C. PCR products were visualized on 1% agarose gels and the most intense products were then selected for sequencing. The successfully amplified DNAs were purified and sequenced by Genomics BioSci & Tech. CO., Ltd.

### Data Analysis

Barcode of Life Database (BOLD) (http://www.boldsystems.org/) and its statistical tools were mainly used for sequence comparison and species identification. We recognized specimens to the species, genus, and family levels if the similarity values were greater than 99%, 92–99%, and 85–92%, respectively. Secondly, we used our own private COI database in Taiwan Fish Database (http://fishdb.sinica.edu.tw), with a total of 195 families, 981 species and 2,162 records, about one-third of the total Taiwanese fish species, for further comparison. Sequences were aligned using BioEdit [18] software version 6.0.5. Neighbor-joining (NJ) trees of Kimura two-parameter (K2P) distance were created to provide a graphic representation of the pattern of divergence between species [19]. The 1000 bootstrap replications were performed in MEGA 3 software [20]. The K2P genetic distances for defining the species, genus and family levels were based on Ward et al. [17]. The first comparison of the sequences was done in January 2009, and the second comparison was conducted three years later in February 2012, when the COI database was more complete.

The identification rate of each taxonomic category was calculated based on the lowest level that molecular identification could perform. In other words, we deducted those PCR-failed samples and those specimens which could not be identified to family level. Then the accuracy of larval fish identification of families, genera and species among the five different laboratories was calculated separately in two different ways: one by treating a blank answer (i.e. unidentified) as incorrect, and the other by skipping blank/unidentified answers in the calculation.

## Results

### Species Identification by Barcoding

Among a total of 100 specimens, 12 samples failed PCR for COI and could not be identified to family level. Of the remaining 88 that had successful PCR, an additional sample (No. 32) could not be identified to family level due to similarity value <84%, although its morphology is similar to Gobiosocidae. This left a total of 87 families, 79 genera and 69 species that could be used to calculate the accuracy of larval fish identification in three taxonomic categories (family, genus and species) among the five labs. In other words, 8 specimens could be identified to only the family level, 10 specimens to only the genus level, and 69 specimens to the species level.

Table 1 shows the increase in identifications between 2009 and 2012 (up from 84 families, 73 genera and 56 species among the 100 morphotypes in 2009). Some species which initially could be identified to only the family or genus level can now be recognized to the species level. In other words, the more complete the COI database, the more larval fishes can be identified to the species level.

**Table 1:** DNA barcoding results for 100 larval fish specimens in two different years.

Date forcomparison	Family	Genus	Species	Null	PCR failure
2009/1/1	84	73	56	4	12
2012/5/1	87	79	69	1	12

### Accuracy of Morphological Identification

The average accuracy rates of the five laboratories were quite low: 71.3–87.9% (average 80.1%) for the family level, 15.2–72.8% (average 41.1%) for the genus level, and 2.9–34.1% (13.5%) for the species level. If the results marked as “unidentified” were excluded from the calculations, the rates went up to 50.7–92.3% (average 75.4%) and 25.0–87.5% (average 43.7%) for the genus and species levels, respectively (Table 2). Thus, we suggest that larval fish identification should be more conservative; i.e., when in doubt, it is better to key only to the family and not to the genus or species level. DNA barcoding is useful for checking the accuracy of traditional larval fish identification among different taxonomists and remains one of the best methods to confirm larval fish species identification.

**Table 2:** The correctness of larval fish identification among the five labs (values in parentheses represent calculations skipping blank/unidentified answers).

Lab code	Family(n = 87) %	Genus(n = 79) %	Species(n = 69) %
A	79.3	34.2 (69.2)	10.1 (30.4)
B	87.9	72.8 (73.7)	34.1 (35.6)
C	75.9	15.2 (92.3)	2.9 (40.0)
D	71.3	45.6 (50.7)	10.1 (25.0)
E	86.2	38.0 (90.9)	10.1 (87.5)
Average	80.1	41.1 (75.4)	13.5 (43.7)

## Discussion

Our study results clearly revealed that the accuracy of using traditional morphological identification on fish larvae was quite low and varied among different taxonomists and laboratories. Because we have promised all five labs that helped with morphological identification that we would keep confidential which label (A to E) belongs to which lab, we cannot discuss and compare the capabilities and skills of the five labs here. The lesson we learned from this study is that larval fish identification using traditional morphological characters should be more conservative, and it is better to identify only to the family or genus level, and not to the species level, in order to avoid the probability of incorrect species identification (66%–97%).

**Figure 1:**
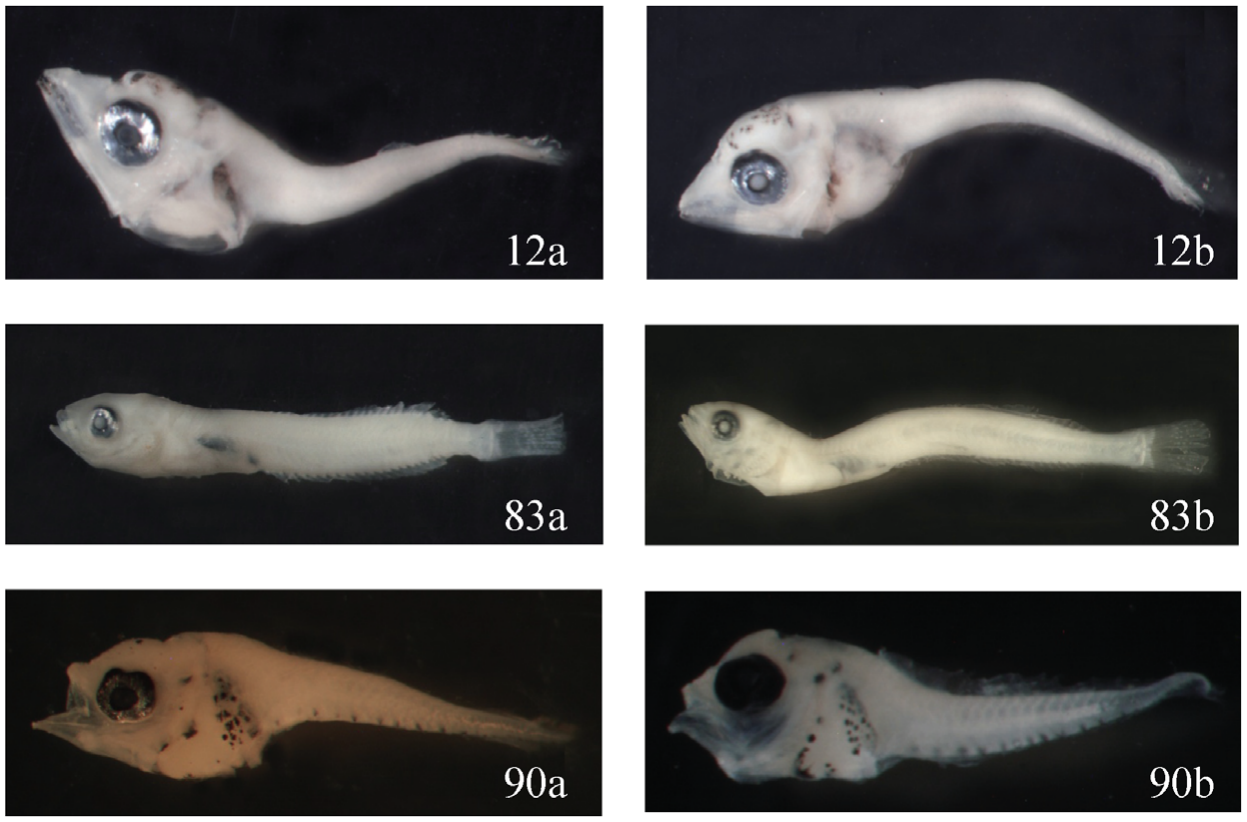
The two specimens of three morphotypes (No. 12, 83, and 90) were identified as different species or families, but were proven to be identical species through barcoding. Shown here: *Katsuwonus pelamis* 4.8 and 4.2 mm SL (12a and 12b); Tripterygiidae 9.5 and 9.3 mm (83a and 83b); and *Abudefduf vaigiensis* 2.8 and 3.0 mm (90a and 90b).

COI barcodes are essential to verify the species identification of fish larvae, especially of morphologically similar species. For example, among the 30 morphotypes that have “reference specimens,” Lab B identified the two specimens of No. 12 and No. 83 as two different species in the same family, and the two specimens of No. 90 to be in two different families (Fig. 1). However, the two specimens of all three morphotypes proved to be the same species through barcoding.

In addition, COI barcodes can help verify the species identification of voucher specimens while identifying larval fishes. For example, No. 1 should be *Decapterus macarellus* (99.36%) when compared with BOLD, but only *D.* sp. (ASIZP 0804324–26) when compared with Taiwan Fish Database. After we re-examined the specimen, it proved to be *D. macarellus.*

**Figure 2:**
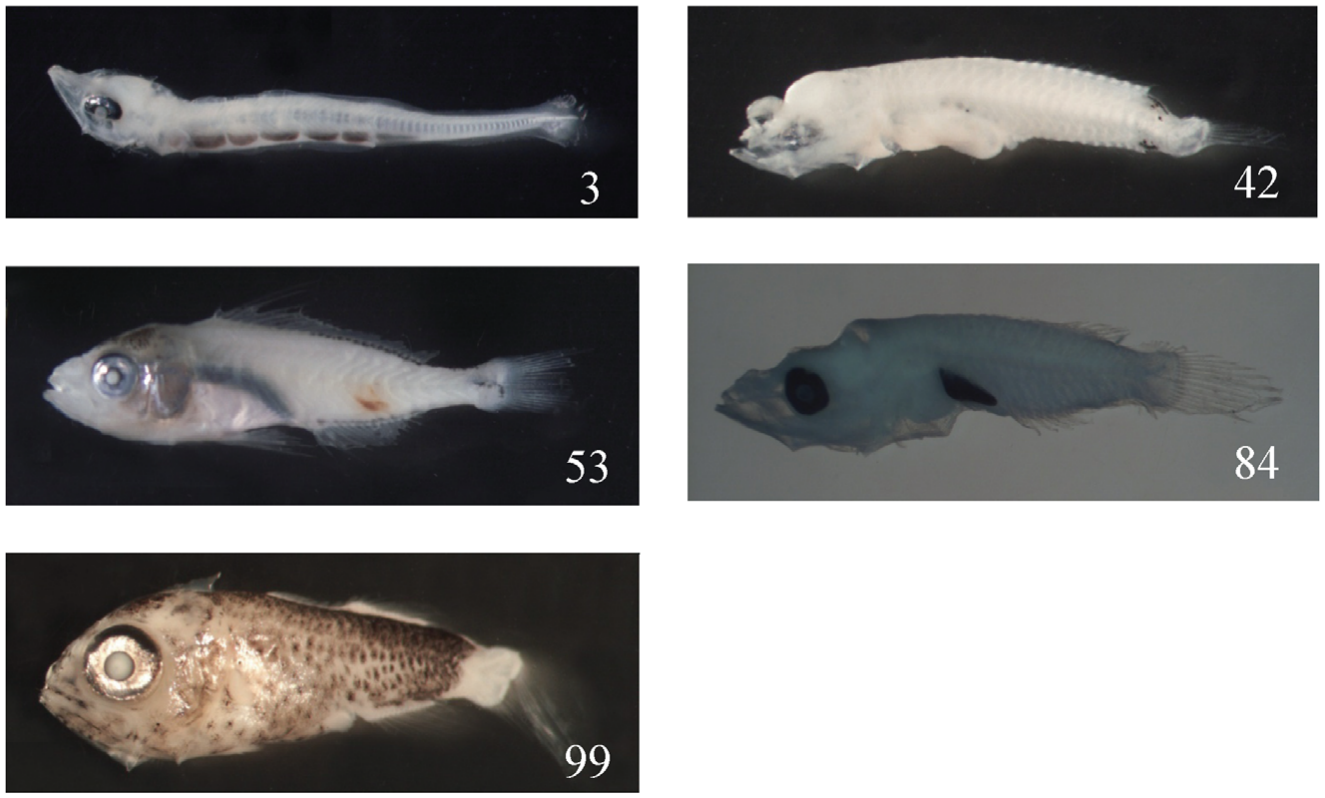
New records can be found by barcoding larval fishes, such as *Sudis hyaline* 8.0 mm SL (No. 3); *Pseudojuloides severnsi* 5.0 mm (No. 42); *Pterocaesio tessellate* 16.0 mm (No. 53); *Cirrhilabrus katherinae* 4.8 mm (No. 84); and *Scombrops gilberti* 6.3 mm (No. 99).

Barcodes can also help discover new records of fishes; in this experiment, we discovered five new fish records in Taiwan. They are: *Sudis hyaline* of No. 3 (99.84%); *Pseudojuloides severnsi* of No. 42 (99.51%); *Pterocaesio tessellate* of No. 53 (99.69%); *Cirrhilabrus katherinae* of No. 84 (99.84%); and *Scombrops gilberti* of No. 99 (99.84%) (Fig. 2). But it is also possible they were misidentified in barcode databases, unless their cataloged specimens were re-examined by qualified fish taxonomists.

**Figure 3:**
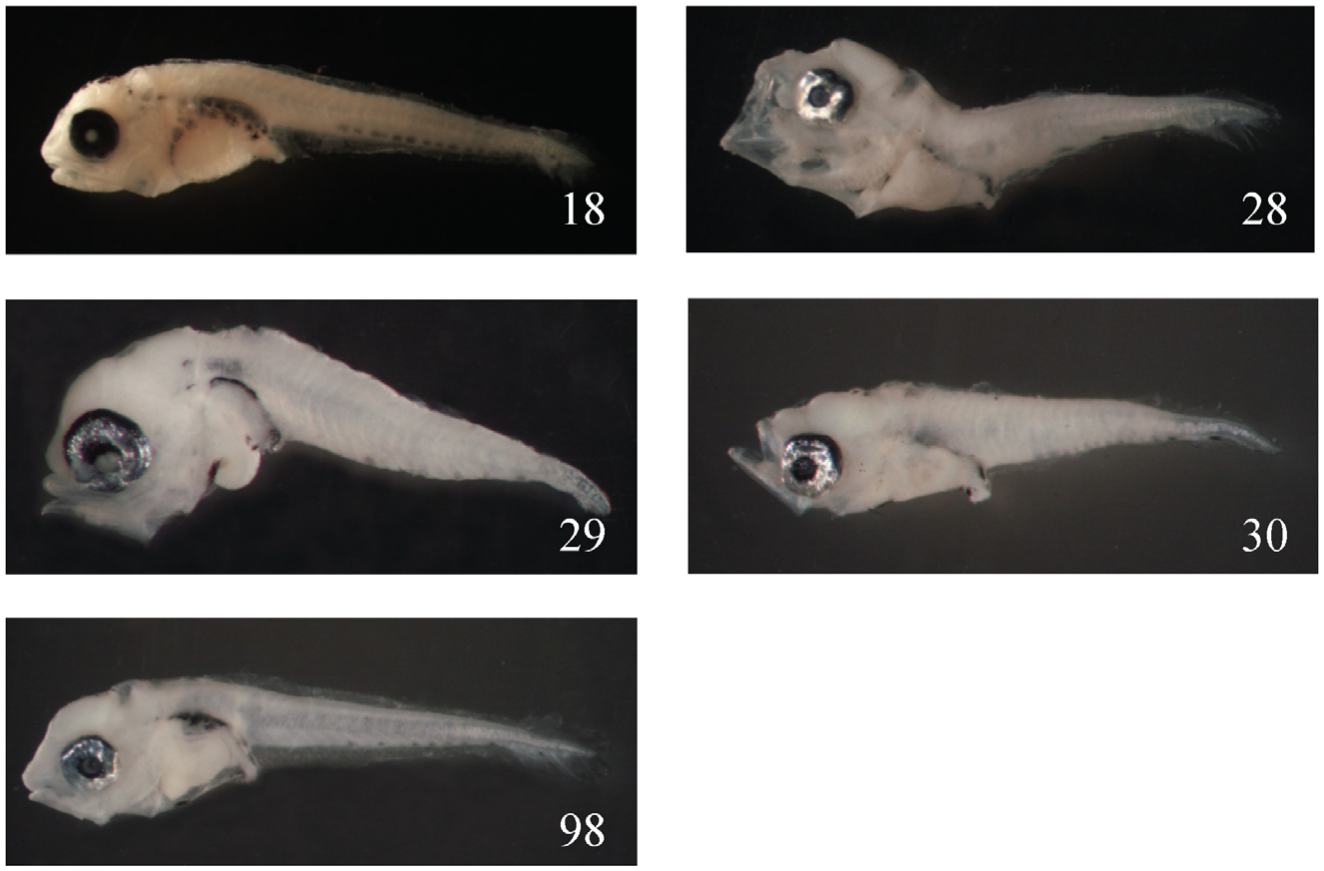
The morphological characters in Sparidae larval fishes are easy to be misidentified. Shown here are *Evynnis cardinalis* 5.5, 4.4, 3.3, and 4.8 mm SL (No. 18, 28, 30, and 98, respectively) and Sparidae 2.6 mm (No. 29).

**Figure 4:**
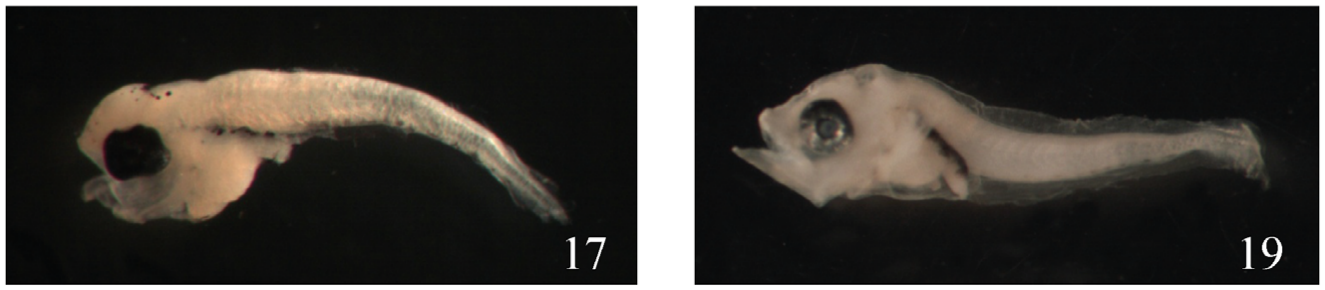
The morphological characters in Scorpaenidae larval fishes are easy to be misidentified. Shown here are *Sebastapistes strongia* 2.2 mm SL (No. 17) and *Sebastiscus marmoratus* 4.3 mm (No. 19).

**Figure 5:**
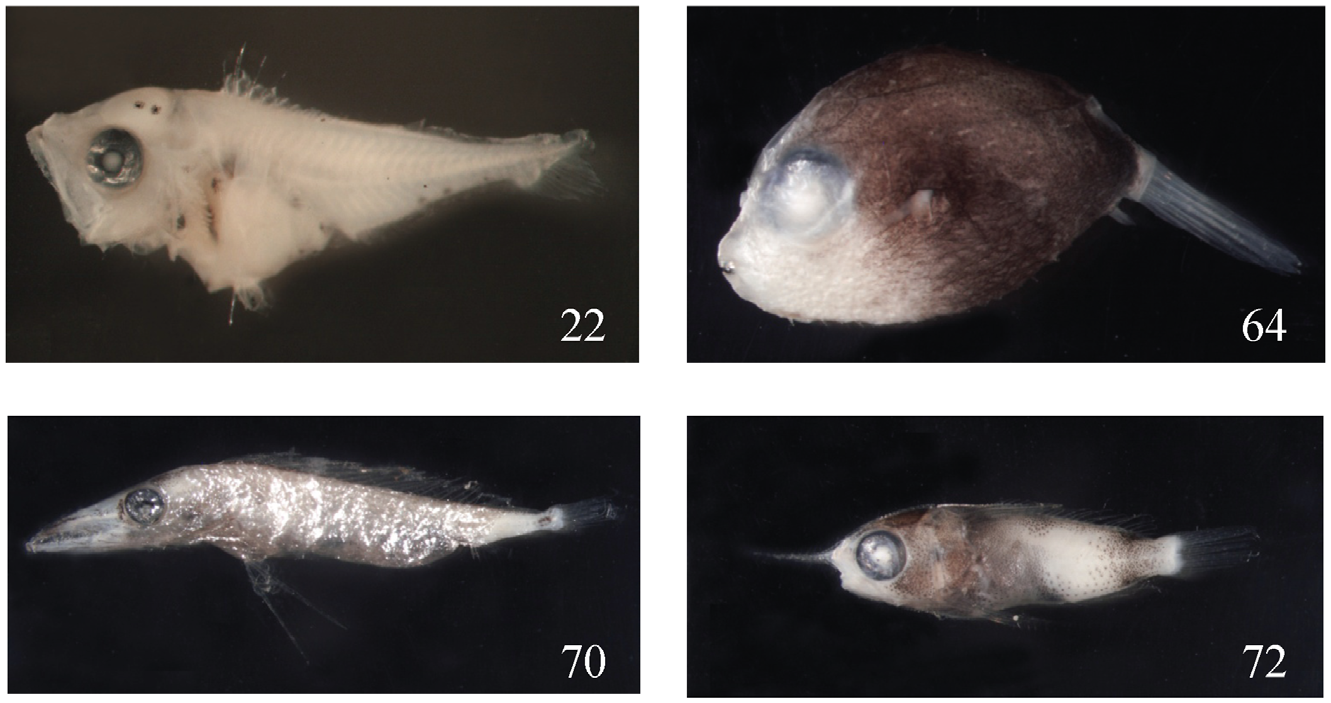
Some families can be easily misidentified as other families: *Caprodon schlegelii* 5.7 mm SL (No. 22); *Canthigaster rivulata* 9.0 mm (No. 64); *Gempylidae* 18.0 mm (No. 70); and *Hoplolatilus* sp. 15.0 mm (No. 72).

From this experiment, using the barcoding method we can infer which families are easier and which are more difficult to be identified. The easily misidentified families are: (1) Sparidae (No. 18, 28, 29, 30, 98), all in preflexion stages without obvious diagnostic characters, misidentified as Scorpaenidae, Scombridae, Haemulidae, Terapontidae, etc. (Fig. 3); (2) Scorpaenidae (No. 17 and 19), in preflexion, misidentified as Scombridae, Sparidae, etc. (Fig. 4); (3) Serranidae (No. 22), misidentified as Lutjanidae because of the similar body shape and the extension of the second DF spine and first VF spine; (4) Malacanthidae (No. 72), misidentified as Holocentridae, because they share the same well-developed and extended nostril spine; (5) Tetraodontidae (No. 64), misidentified as Diodontidae; and (6) Gempylidae (No. 70), misidentified as *Trichurus* (Fig. 5).

**Figure 6:**
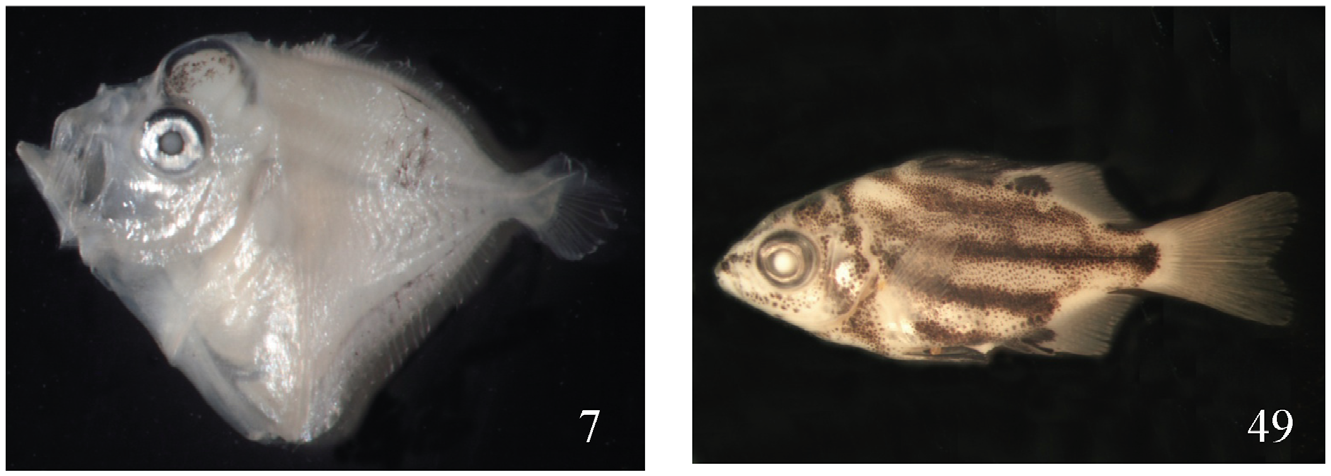
Only two specimens were identified correctly by all five labs: *Mene maculata* 4.5 mm SL (No. 7) and *Microcanthus strigatus* 15.0 mm (No. 49).

On the other end, the easiest identified families or species are *Mene maculate* (No. 7) and *Microcanths strigatus* (No. 49) (Fig. 6). Because they all have peculiar diagnostic characters, all five labs could identify them to species level correctly. Although barcoding remains one of the best methods to confirm species identification, molecular identification of larval fishes still has some deficiencies and bottlenecks. The most serious problem is the COI database being incomplete, especially for those non-economic and difficult to identify families. Eight specimens could be identified only to the family level (similarity values: 85–89%): Ophichthidae (No. 6), Engraulidae (No. 8), Belonidae (No. 15), Sparidae (No. 29), Notocheiridae (No. 44), Malacanthidae (No. 71), Tripterygiidae (No. 83), and Gobiidae (No. 94). In general, these families belong to groups that are difficult for morphological identification. Consequently, their COI data is not easily accumulated. Similar reasons applied to the 10 specimens which could be identified only to the genus level (92–99%).

The importance of having a more complete and accurate barcode database for larval fish molecular identification can also be demonstrated in this paper when we compared with the database again after three years (2009 vs. 2012). Three more families, 6 more genera, and 13 more species found matches simply because the COI database was becoming more complete and reliable for species identification. For example, No. 16 and 17 were identified as *Sebastapistes strongia* earlier (similarity value: 98.72% and 99.84%) but later changed to *Parascorpaena mossambica* (99.83 and 99.84%), probably because the species identification in the database was corrected.

Having a more complete COI database for all fish species can make fish egg and fish larva identification more successful. The DNA barcoding method was developed in 2003 [21] and began to be applied to fishes in the earliest stage. The first country that established a COI databank for fishes was Australia [17]. The Fish-BOL campaign (http://www.fishbol.org) was conceived in 2004 [22], and as of Oct 2012 there have been 136,758 COI sequences belonging to 12,909 fish species that have been deposited in BOLD.

As to the 12 specimens that failed PCR, it could be a quality problem with the tissue sample, the need for a special primer or some other technical issue. Under the circumstances, morphological identification of larval fishes is still necessary to compensate for the failure of molecular identification. However, it is suggested to identify only to the family or genus level, and not to the species level to avoid identification errors. Taking good photos or good drawings of larval fish specimens and depositing more cryobank or alcohol specimens for future identification are also recommended.

## Supporting Information

Table S1 **The results of 100 experimental larval fish specimens identified morphologically by five laboratories (A–E) and DNA barcoding by Academia Sinica (2^nd^ column).** In this table, “NA” means unidentified or no answer was provided by the lab; “NF” means no family could be found by COI comparison; “NP” means PCR failure; an asterisk (*) means not recorded in Taiwan; bold means incorrect answers.
(DOC)
