# Supplementary material for: Evaluating the Accuracy of Morphological Identification of Larval Fishes by Applying DNA Barcoding
Source: PLoS One. 2013 Jan 31;8(1):e53451. doi: 10.1371/journal.pone.0053451 (PMC3561387; doi:10.1371/journal.pone.0053451)
Supplement: Table S1 — The results of 100 experimental larval fish specimens identified morphologically by five laboratories (A–E) and DNA barcoding by Academia Sinica (2nd column). In this table, “NA” means unidentified or no answer was provided by the lab; “NF” means no family could be found by COI comparison; “NP” means PCR failure; an asterisk (*) means not recorded in Taiwan; bold means incorrect answers. (DOC) [file pone.0053451.s001.doc]

Table S1. The results of 100 experimental larval fish specimens identified morphologically by five laboratories (A–E) and DNA barcoding by Academia Sinica (2^nd^ column). In this table, “NA” means unidentified or no answer was provided by the lab; “NF” means no family could be found by COI comparison; “NP” means PCR failure; an asterisk (*) means not recorded in Taiwan; bold means incorrect answers.

| No. | Barcode  Identification | Similarity  (%) | N | A | B | C | D | E |
| --- | --- | --- | --- | --- | --- | --- | --- | --- |
| 67 | Acanthuridae  *Naso lopezi* | 99.37 | 2 | Acanthuridae  *Naso* sp. | Acanthuridae  *Naso* ***unicornis*** | Acanthuridae  *Naso* sp. | Acanthuridae  *Naso* sp. | Acanthuridae  *Naso* sp. |
| 66 | Acropomatidae  *Synagrops* sp. | 96.34 | 1 | Acropomatidae  *Synagrops* sp. | Acropomatidae  *Synagrops japonicus* | Acropomatidae | Acropomatidae  *Synagrops* sp. | Acropomatidae |
| 69 | Acropomatidae  *Synagrops japonicus* | 99.84 | 2 | Acropomatidae  *Synagrops* sp. | Acropomatidae  *Synagrops japonicus* | Acropomatidae | Acropomatidae  *Synagrops* sp. | Acropomatidae |
| 80 | Acropomatidae  *Synagrops japonicus* | 99.65 | 1 | Acropomatidae  *Synagrops* sp. | Acropomatidae  *Synagrops japonicus* | Acropomatidae | Acropomatidae  *Synagrops* sp. | Acropomatidae |
| 24 | Ambassidae  *Ambassis* sp. | 92.06 | 1 | Ambassidae  *Ambassis* sp. | Ambassidae  *Ambassis urotaenia* | Ambassidae | Ambassidae  *Ambassis* sp. | Ambassidae  *Ambassis* sp. |
| 93 | Ambassidae  *Ambassis vachellii* | 99.83 | 1 | Ambassidae  *Ambassis* sp. | Ambassidae  *Ambassis* ***urotaenia*** | Ambassidae | Ambassidae  *Ambassis* sp. | Ambassidae  *Ambassis* sp. |
| 9 | Apogonidae  *Archamia bleekeri* | 99.21 | 2 | Apogonidae  ***Apogon lineatus*** | Apogonidae  *Archamia bleekeri* | Apogonidae | Apogonidae  ***Apogon*** sp. | Apogonidae  ***Apogon*** sp. |
| 62 | Apogonidae  *Apogon* sp. | 94.90 | 1 | Apogonidae | Apogonidae  *Apogon erythrinus* | Apogonidae | Apogonidae  *Apogon* sp. | Apogonidae  *Apogon* sp. |
| 56 | Apogonidae  *Rhabdamia* sp. | 97.97 | 1 | Apogonidae | Apogonidae  *Rhabdamai gracilis* | Apogonidae | **Caesionidae *Gymnocaesio gymnoptera*** | Apogonidae |
| 74 | Balistidae  *Sufflamen bursa* | 99.84 | 1 | Balistidae | Balistidae  *Sufflamen* ***fraetus*** | **Monacanthidae** | Balistidae  *Sufflamen* sp. | Balistidae |
| 15 | Belonidae | 88.81 | 1 | Belonidae | Belonidae  *Strongylura strongylura* | Belonidae | Belonidae  *Hemiramphus* sp. | Belonidae |
| 85 | Berycidae  *Centroberyx druzhinini* | 100 | 1 | NA | Berycidae  ***Beryx splendens*** | NA | Berycidae  ***Beryx*** sp. | Berycidae |
| 43 | Blenniidae  *Andamia reyi* | 99.84 | 1 | Blenniidae | Blenniidae  *Andamia reyi* | Blenniidae | Tripterygiidae | Blenniidae |
| 53 | Caesionidae  *Pterocaesio tessellata ** | 99.69 | 1 | Caesionidae  ***Caesio*** sp. | Caesionidae  *Pterocaesio* ***digramma*** | Lutjanidae | Caesionidae ***Gymnocaesio gymnoptera*** | Caesionidae  ***Caesio*** sp. |
| 1 | Carangidae  *Decapterus macarellus* | 99.36 | 2 | Carangidae  *Decapterus macarellus* | Carangidae  *Decapterus macarellus* | Carangidae  *Decapterus* sp. | Carangidae  *Decapterus* sp. | Carangidae  *Decapterus* sp. |
| 97 | Carangidae  *Trachurus japonicus* | 99.67 | 1 | Carangidae  *Trachurus japonicus* | Carangidae  ***Elagatis bipinnulata*** | Carangidae  *Trachurus japonicus* | **Platycephalidae** | Carangidae |
| 96 | Cheilodactylidae *Cheilodactylus quadricornis* | 99.63 | 1 | **Kyphosidae**  ***Girella*** sp. | Cheilodactylidae ***Goniistius zonatus*** | **Kyphosidae** | **Mugilidae**  ***Girella* sp.** | **Mugilidae** |
| 68 | Diretmidae  *Diretmoides pauciradiatus* | 99.53 | 2 | Diretmidae | Diretmidae  ***Diretmus argenteus*** | Diretmidae | Diretmidae  ***Diretmichthys parini*** | Diretmidae |
| 8 | Engraulidae | 86.5 | 2 | Engraulidae  *Engraulis japonicus* | Engraulidae *Encrasicholina heteroloba* | Engraulidae | Engraulidae  *Coilia mystus* | Engraulidae |
| 70 | Gempylidae  *Nesiarchus* sp. | 94.31 | 1 | **Trichiuridae** | Gempylidae ***Promethichthys*** *prometheus* | **Trichiuridae** | Gempylidae  *Nesiarchus nasutus* | Gempylidae |
| 75 | Gempylidae  *Neoepinnula* sp. | 96.26 | 1 | Gempylidae | Gempylidae  *Neoepinnula orientalis* | Gempylidae | Gempylidae  ***Ruvettus*** *pretiosus* | Gempylidae |
| 76 | Gempylidae  *Nealotus tripes* | 99.05 | 2 | Gempylidae | Gempylidae  *Nealotus tripes* | Gempylidae | Gempylidae  *Nealotus tripes* | Gempylidae |
| 23 | Gerreidae  *Gerres filamentosus* | 99.84 | 1 | Gerreidae  *Gerres* ***abbreviatuis*** | Gerreidae  *Gerres* ***erythrourus*** | Gerreidae  *Gerres* ***abbreviatuis*** | Gerreidae  *Gerres* sp. | Gerreidae  *Gerres* sp. |
| 82 | Gobiidae  *Oxyurichthys* sp. | 97.78 | 2 | Gobiidae | Gobiidae  *Oxyurichthys ophthalmonema* | Gobiidae | **Ptereleotridae *Parioglossus formosus*** | Gobiidae |
| 36 | Gobiidae  *Psammogobius biocellatus* | 99.83 | 1 | Gobiidae | Gobiidae | Gobiidae | **Ammodytidae *Embolichthys mitsukurii*** | Gobiidae |
| 94 | Gobiidae | 87.78 | 2 | Gobiidae | Gobiidae | Gobiidae | Gobiidae  *Redigobius bikolanus* | Gobiidae |
| 95 | Gonorynchidae  *Gonorynchus abbreviatus* | 99.84 | 1 | Gonorynchidae *Gonorynchus abbreviatus* | Gonorynchidae *Gonorynchus abbreviatus* | Gonorynchidae | Gonorynchidae *Gonorynchus* ***greyi*** | Gonorynchidae *Gonorynchus abbreviatus* |
| 27 | Haemulidae  *Pomadasys quadrilineatus* | 99.84 | 1 | **Percichthyidae** | **Percichthyidae *Lateolabrax japonicus*** | **Percichthyidae** | **Gerreidae**  ***Gerres abbreviatus*** | **Apogonidae** |
| 10 | Istiophoridae  *Istiophorus platypterus* | 100 | 2 | Istiophoridae | Istiophoridae  *Istiophorus platypterus* | Istiophoridae | Istiophoridae  ***Tetrapturus angustirostris*** | **Holocentridae** |
| 49 | Kyphosidae  *Microcanthus strigatus* | 99.22 | 1 | Kyphosidae  *Microcanthus strigatus* | Kyphosidae  *Microcanthus strigatus* | Kyphosidae | Kyphosidae  *Microcanthus strigatus* | Kyphosidae  *Microcanthus strigatus* |
| 84 | Labridae  *Cirrhilabrus katherinae ** | 100 | 1 | Labridae | Labridae  *Cirrhilabrus* ***temminckii*** | Labridae | Labridae | Labridae |
| 42 | Labridae  *Pseudojuloides severnsi ** | 99.51 | 1 | Labridae  ***Pseudolabrus***sp. | Labridae  ***Halichoeres tenuispinnis*** | Labridae | Labridae | Labridae |
| 50 | Labridae  *Pseudolabrus eoethinus* | 100 | 1 | **Percichthyidae** | Labridae  *Pseudolabrus eoethinus* | **Percichthyidae** | **Aulopidae**  ***Aulopus* sp.** | **Aulopidae**  ***Aulopus* sp.** |
| 59 | Lethrinidae  *Lethrinus nebulosus* | 100 | 1 | Lethrinidae | Lethrinidae  *Lethrinus* ***lentjan*** | Lethrinidae | Lethrinidae  *Lethrinus* sp. | Lethrinidae  *Lethrinus* sp. |
| 54 | Lethrinidae  *Lethrinus olivaceus* | 100 | 1 | Lethrinidae | Lethrinidae  *Lethrinus* ***haematopterus*** | Lethrinidae | **Gerreidae**  ***Gerres*** sp. | Lethrinidae  *Lethrinus* sp. |
| 55 | Lethrinidae  *Lethrinus olivaceus* | 99.53 | 1 | Lethrinidae | Lethrinidae  *Lethrinus* ***harak*** | Lethrinidae | Lethrinidae  *Lethrinus* sp. | Lethrinidae |
| 79 | Lutjanidae  *Etelis coruscans* | 99.84 | 2 | Lutjanidae | Lutjanidae  ***Pristipomoides multidens*** | Lutjanidae | Lutjanidae  *Etelis* sp. | Lutjanidae  *Etelis coruscans* |
| 92 | Lutjanidae  *Lutjanus fulviflamma* | 100 | 1 | Lutjanidae | Lutjanidae  *Lutjanus* ***monostigma*** | Lutjanidae | Lutjanidae  *Lutjanus* ***argentimaculatus*** | Lutjanidae  *Lutjanus* sp. |
| 71 | Malacanthidae | 88.75 | 2 | Malacanthidae | Malacanthidae  *Caulolatilus princeps* | Malacanthidae | Malacanthidae *Branchiostegus* sp. | Malacanthidae |
| 72 | Malacanthidae  *Hoplolatilus* sp. | 98.04 | 1 | **Holocentridae** | Malacanthidae *Hoplolatilus fronticinctus* | **Holocentridae** | **Holocentridae *Sargocentron* sp.** | **Holocentridae** |
| 78 | Malacanthidae  *Hoplolatilus marcosi* | 100 | 1 | Malacanthidae | Malacanthidae ***Branchiostegus japonicus*** | Malacanthidae | Malacanthidae ***Branchiostegus*** sp. | Malacanthidae |
| 7 | Menidae  *Mene maculata* | 99.84 | 2 | Menidae  *Mene maculata* | Menidae  *Mene maculata* | Menidae  *Mene maculata* | Menidae  *Mene maculata* | Menidae  *Mene maculata* |
| 35 | Mullidae  *Upeneus japonicus* | 99.84 | 1 | Mullidae | Mullidae  *Upeneus japonicus* | Mullidae | Mullidae  *Upeneus* sp. | Mullidae |
| 44 | Notocheiridae | 85.15 | 2 | **Atherinidae** | **Atherinidae**  *Hypoatherina valenciennei* | **Atherinidae** | Notocheiridae  *Iso* sp. | **Atherinidae** |
| 6 | Ophichthidae | 87.64 | 1 | Ophichthidae | Ophichthidae  *Ophichthus* sp. | Ophichthidae | Ophichthidae  *Ophichthinae* sp. | Ophichthidae |
| 3 | Paralepididae  *Sudis hyalina* *** | 99.84 | 1 | Paralepididae  *Sudis* sp. | Paralepididae  *Sudis* ***atrox*** | Paralepididae  *Sudis* sp. | Paralepididae  *Sudis* ***atrox*** | Paralepididae |
| 60 | Pempheridae  *Parapriacanthus ransonneti* | 95.66 | 1 | Apogonidae | Pempheridae *Parapriacanthus ransonneti* | Apogonidae | Pempheridae *Parapriacanthus ransonneti* | Pempheridae |
| 26 | Pempheridae  *Pempheris schwenkii* | 100 | 1 | Pempheridae | Pempheridae  *Pempheris* ***xanthoptera*** | Pempheridae | Pempheridae  *Pempheris* ***xanthoptera*** | Pempheridae  *Pempheris* sp. |
| 34 | Pempheridae  *Pempheris schwenkii* | 100 | 2 | Pempheridae | Pempheridae  *Pempheris* ***nyctereutes*** | Pempheridae | Pempheridae  *Pempheris* ***xanthoptera*** | Pempheridae  *Pempheris* sp. |
| 73 | Pomacanthidae  *Centropyge ferrugata* | 99.84 | 2 | Pomacanthidae | Pomacanthidae  *Centropyge* ***tibicen*** | Pomacanthidae | Pomacanthidae | Pomacanthidae |
| 21 | Pomacentridae  *Abudefduf vaigiensis* | 100 | 1 | Pomacentridae  *Abudefduf* sp. | Pomacentridae  *Abudefduf vaigiensis* | Pomacentridae  *Abudefduf* sp. | Pomacentridae  *Abudefduf* sp. | Pomacentridae  *Abudefduf* sp. |
| 90 | Pomacentridae  *Abudefduf vaigiensis* | (a)100  (b)100 | 2 | Mullidae | Scombridae  (a)***Scomber australasicus*** (b)*Abudefduf vaigiensis* | Mullidae | **Scombridae *Scomberomorus guttatus*** | Pomacentridae  *Abudefduf s*p. |
| 2 | Pomacentridae  *Chromis okamurai* | 99.68 | 1 | Pomacentridae  *Chromis* sp. | Pomacentridae  *Chromis* ***notata*** | Pomacentridae | Pomacentridae ***Pomacentrus*** sp. | Pomacentridae  *Chromis* sp. |
| 100 | Pomacentridae  *Chromis okamurai* | 99.67 | 1 | Pomacentridae  *Chromis* sp. | Pomacentridae  *Chromis* ***notata*** | Pomacentridae  *Chromis* sp. | Pomacentridae  *Chromis* sp. | Pomacentridae  *Chromis* sp. |
| 57 | Pomacentridae  *Chromis viridis* | 100 | 1 | Pomacentridae | Pomacentridae  *Chromis viridis* | Pomacentridae | Pomacentridae  *Chromis* ***fumea*** | Pomacentridae  *Chromis viridis* |
| 58 | Pomacentridae *Neoglyphidodon nigroris* | 99.84 | 1 | Nemipteridae  ***Scolopsis*** sp. | Pomacentridae *Neoglyphidodon nigroris* | Nemipteridae | Pomacentridae *Neoglyphidodon nigroris* | Pomacentridae *Neoglyphidodon nigroris* |
| 52 | Pomacentridae *Plectroglyphidodon lacrymatus* | 99.84 | 1 | Pomacentridae | Pomacentridae *Plectroglyphidodon* ***leucozonus*** | Pomacentridae | Pomacentridae  ***Chromis*** sp. | Pomacentridae *Plectroglyphidodon lacrymatus* |
| 89 | Scombridae  *Auxis rochei* | 100 | 1 | Scombridae  *Auxis* sp. | Scombridae  *Auxis rochei* | Scombridae  *Auxis* sp. | Scombridae ***Scomberomorus guttatus*** | Scombridae |
| 14 | Scombridae  *Euthynnus affinis* | 100 | 1 | Scombridae  *Euthynnus affinis* | Scombridae  *Euthynnus affinis* | Scombridae | Scombridae  ***Thunnus obesus*** | Scombridae |
| 12 | Scombridae  *Katsuwonus pelamis* | (a) 100  (b) 99.84 | 2 | Scombridae  ***Euthynnus affinis*** | Scombridae  (a)***Auxis thazard***  (b)***Euthynnus affinis*** | Scombridae  ***Thunnus*** sp. | Scombridae  ***Thunnus obesus*** | Scombridae |
| 31 | Scombridae  *Scomber japonicus* | 100 | 1 | Scombridae  *Scomber* ***australasicus*** | Scombridae  *Scomber* ***australasicus*** | Scombridae  *Scomber* ***australasicus*** | **Mullidae**  ***Upeneus* sp.** | Scombridae |
| 37 | Scombridae  *Scomber japonicus* | 99.81 | 1 | **Scorpaenidae**  ***Sebastiscus marmoratus*** | Scombridae  *Scomber japonicus* | **Scorpaenidae** | **Mullidae**  ***Upeneus* sp.** | **Mullidae** |
| 99 | Scombropidae  *Scombrops gilberti ** | 99.84 | 1 | Scombropidae  *Scombrops* ***boops*** | Scombropidae  *Scombrops* ***boops*** | Scombropidae  *Scombrops* ***boops*** | Scombropidae  *Scombrops* ***boops*** | Scombropidae  *Scombrops* ***boops*** |
| 16 | Scorpaenidae  *Sebastapistes strongia* | 99.84 | 1 | Scorpaenidae | Scorpaenidae ***Scorpaenodes littoralis*** | Scorpaenidae | Scorpaenidae ***Scorpaenodes*** sp. | Scorpaenidae |
| 17 | Scorpaenidae  *Sebastapistes strongia* | 99.83 | 1 | Scorpaenidae  ***Sebastiscus marmoratus*** | **Scombridae**  ***Scomber japonicus*** | **Sparidae** | **Ambassidae**  ***Ambassis* sp.** | **Sparidae** |
| 38 | Scorpaenidae  *Sebastiscus albofasciatus* | 99.84 | 2 | Scorpaenidae  *Sebastiscus* ***marmoratus*** | Scorpaenidae  *Sebastiscus* ***marmoratus*** | Scorpaenidae | **Haemulidae**  ***Pomadasys* sp**. | Scorpaenidae |
| 39 | Scorpaenidae  *Sebastiscus albofasciatus* | 100 | 1 | Scorpaenidae  *Sebastiscus* ***marmoratus*** | Scorpaenidae  *Sebastiscus* ***marmoratus*** | Scorpaenidae | Scorpaenidae  *Sebastiscus* sp. | Scorpaenidae |
| 40 | Scorpaenidae  *Sebastiscus albofasciatus* | 100 | 1 | Scorpaenidae  *Sebastiscus* ***marmoratus*** | Scorpaenidae  *Sebastiscus* ***marmoratus*** | Scorpaenidae | Scorpaenidae  *Sebastiscus* sp. | Scorpaenidae |
| 19 | Scorpaenidae  *Sebastiscus marmoratus* | 100 | 1 | Scorpaenidae  *Sebastiscus marmoratus* | **Apogonidae**  ***Apogon* sp.** | Scorpaenidae | **Lutjanidae**  ***Lutjanus* sp.** | **Scombridae** |
| 22 | Serranidae  *Caprodon schlegelii* | 100 | 1 | Serranidae | **Lutjanidae**  ***Lutjanus* sp.** | Serranidae | **Lutjanidae**  ***Symphorus nematophorus*** | Serranidae |
| 65 | Serranidae  *Pseudanthias squamipinnis* | 99.21 | 1 | Serranidae | Serranidae  *Pseudanthias squamipinnis* | Serranidae | **Acropomatidae *Acropoma japonicum*** | **Acropomatidae** |
| 20 | Serranidae  *Pseudanthias squamipinnis* | 99.84 | 1 | Serranidae | Serranidae  ***Sacura margaritacea*** | Serranidae | Serranidae  ***Plectranthias*** sp. | Serranidae |
| 13 | Sillaginidae  *Sillago sihama* | 99.34 | 2 | Sillaginidae  *Sillago* ***japonica*** | Sillaginidae  *Sillago* ***japonica*** | Sillaginidae  *Sillago* sp. | Sillaginidae  *Sillago* sp. | Sillaginidae  *Sillago* sp. |
| 25 | Sparidae  *Acanthopagrus berda* | 100 | 2 | Sparidae  *Acanthopagrus* ***schlegelii*** | Sparidae  *Acanthopagrus* ***schlegelii*** | Sparidae | Sparidae  *Acanthopagrus berda* | Sparidae  *Acanthopagrus* sp. |
| 18 | Sparidae  *Evynnis cardinalis* | 99.69 | 2 | **Scombridae**  ***Scomber australasicus*** | **Scombridae**  ***Scomber australasicus*** | **Scombridae** | **Mullidae**  ***Parupeneus* sp.** | Sparidae  *Evynnis* sp. |
| 28 | Sparidae  *Evynnis cardinalis* | 100 | 2 | **Scorpaenidae**  ***Sebastiscus marmoratus*** | **Caesionidae**  ***Pterocaesio digramma*** | **Scorpaenidae** | **Sciaenidae** | Sparidae  *Evynnis* sp. |
| 30 | Sparidae  *Evynnis cardinalis* | 99.84 | 1 | **Scorpaenidae**  ***Sebastiscus marmoratus*** | **Haemulidae *Parapristipoma trilineatum*** | **Scorpaenidae** | **Terapontidae**  ***Terapon theraps*** | **Haemulidae** |
| 98 | Sparidae  *Evynnis cardinalis* | 99.84 | 1 | **Scorpaenidae**  ***Sebastiscus marmoratus*** | Sparidae  ***Acanthopagrus latus*** | **Scorpaenidae** | **Terapontidae**  ***Terapon theraps*** | **Scorpaenidae** |
| 29 | Sparidae | 88.68 | 1 | **Scorpaenidae**  ***Sebastiscus marmoratus*** | **Apogonidae**  ***Apogon* sp.** | **Scorpaenidae** | A  **Percoidei (鱸亞目)** | Sparidae |
| 4 | Sphyraenidae  *Sphyraena barracuda* | 99.68 | 1 | Sphyraenidae  *Sphyraena* sp. | Sphyraenidae  *Sphyraena barracuda* | Sphyraenidae | Sphyraenidae  *Sphyraena* sp. | Sphyraenidae  *Sphyraena* sp. |
| 77 | Symphysanodontidae *Symphysanodon katayamai* | 99.84 | 1 | Symphysanodontidae | Symphysanodontidae *Symphysanodon katayamai* | Symphysanodontidae | Symphysanodontidae *Symphysanodon katayamai* | Symphysanodontidae *Symphysanodon* sp. |
| 46 | Terapontidae  *Pelates quadrilineatus* | 99.68 | 1 | **Kuhliidae** | Terapontidae  *Pelates quadrilineatus* | NA | **Sillaginidae**  ***Sillago* sp.** | Terapontidae  *Pelates* sp. |
| 64 | Tetraodontidae  *Canthigaster rivulata* | 99.47 | 2 | **Diodontidae** | **Diodontidae**  ***Diodon holocanthus*** | **Diodontidae** | Tetraodontidae  ***Arothron immaculatus*** | Tetraodontidae |
| 91 | Tetraodontidae  *Lagocephalus inermis* | 99.83 | 1 | Tetraodontidae | Tetraodontidae *Lagocephalus* ***wheeleri*** | Tetraodontidae | Tetraodontidae *Lagocephalus* sp. | Tetraodontidae |
| 63 | Triacanthodidae  *Tydemania* sp. | 98.13 | 2 | Triacanthodidae | Triacanthodidae *Tydemania navigatoris* | Triacanthodidae | **Balistidae** | Triacanthodidae |
| 83 | Tripterygiidae | (a) 85.19  (b) 85.05 | 2 | Tripterygiidae | Tripterygiidae  (a)*Springerichthys bapturus* (b)*Enneapterygius etheostoma* | Tripterygiidae | Tripterygiidae | Tripterygiidae |
| 86 | Tripterygiidae  *Enneapterygius* sp. | 98.7 | 2 | Tripterygiidae | Tripterygiidae *Enneapterygius* sp. | Tripterygiidae | Tripterygiidae | Tripterygiidae |
| 32 | NF |  | 2 | Gobiesocidae | Gobiesocidae | Gobiesocidae | Ammodytidae *Embolichthys mitsukurii* | Gobiesocidae |
| 5 | NP |  | 2 | Gonostomatidae *Cyclothone alba* | Gonostomatidae *Cyclothone alba* | Gonostomatidae *Cyclothone alba* | Gonostomatidae *Cyclothone* sp. | Gonostomatidae |
| 11 | NP |  | 1 | Hemiramphidae *Hyporhamphus* sp. | Hemiramphidae *Hyporhamphus limbatus* | Hemiramphidae | Hemiramphidae *Hyporhamphus* sp. | Hemiramphidae *Hyporhamphus* sp. |
| 33 | NP |  | 2 | Gobiesocidae | Gobiesocidae  *Lepadichthys frenatus* | Gobiesocidae | Gobiesocidae | Gobiesocidae |
| 41 | NP |  | 1 | Terapontidae  *Terapon jarbua* | Terapontidae  *Terapon jarbua* | Terapontidae | Terapontidae  *Pelates* sp. | Gerreidae |
| 45 | NP |  | 1 | Trichiuridae  *Trichiurus lepturus* | Trichiuridae  *Trichiurus lepturus* | Trichiuridae | Trichiuridae  *Tentoriceps cristatus* | Trichiuridae |
| 47 | NP |  | 1 | Scorpaenidae  *Sebastiscus marmoratus* | Scombridae  *Scomber australasicus* | Scorpaenidae | Pinguipedidae | Sparidae |
| 48 | NP |  | 1 | Scombridae  *Scomber australasicus* | Scombridae  *Auxis thazard* | Scombridae  *Scomber* sp. | Scombridae *Scomberomorus guttatus* | Scombridae |
| 51 | NP |  | 1 | Scorpaenidae  *Sebastiscus marmoratus* | Scorpaenidae  *Sebastiscus marmoratus* | Scorpaenidae | Scorpaenidae  *Sebastiscus* sp. | Scorpaenidae |
| 61 | NP |  | 1 | Scaridae | Nemipteridae  *Nemipterus virgatus* | Scaridae | Scaridae | Scaridae |
| 81 | NP |  | 1 | Tripterygiidae | Gobiidae | Tripterygiidae | Tripterygiidae | Gobiidae |
| 87 | NP |  | 1 | Myctophidae  *Lampadena* sp. | Myctophidae  *Lampadena* sp. | Myctophidae  *Lampadena luminosa* | Myctophidae  *Lampadena* sp. | Myctophidae |
| 88 | NP |  | 1 | Blenniidae | Blenniidae  *Petroscirtes mitratus* | Blenniidae | Blenniidae  *Nemophinae* sp. | Blenniidae |
